# A survey on idiopathic congenital talipes equinovarus (ICTEV) managed by the Ponseti technique at Mulago Hospital - Uganda

**DOI:** 10.11604/pamj.2021.38.397.26560

**Published:** 2021-04-23

**Authors:** Raymond Joseph Malinga, Geoffrey Madewo, Nobert Orwotho, Shafique Pyrali Pirani, Adam Moyosore Afodun, Mustapha Akajewole Masud

**Affiliations:** 1 Department of Anatomy and Cell Biology, Faculty of Health Sciences, Busitema University, Mbale, Uganda,; 2 Department of Orthopedics, Makarere University, Kampala, Uganda,; 3 Department of Orthopedics, Faculty of Medicine, University of British Columbia, Vancouver, Canada,; 4 Department of Human Anatomy, School of Health and Medical Sciences, State University of Zanzibar, Zanzibar, Tanzania

**Keywords:** Ponseti method, midterm evaluation, clinical outcome

## Abstract

**Introduction:**

Ponseti technique is the treatment of choice for idiopathic congential talipes equino varus (ICTEV) since 1950s with excellent treatment outcomes reported worldwide. However, despite the popularity of this technique, Uganda adapted it as a treatment modality for ICTEV in May 2005. Since then, the effectiveness of delivered Ponseti care to children with this very common orthopaedic deformity under the supervision of an orthopaedic surgeon was unknown. The implication of this undertaking was that, satisfactory outcomes would then support the Ministry of Health (MOH)-Uganda´s decision to embrace this mode of treatment and if the outcomes were unsatisfactory, MOH would then consider a policy revision in this regard. To assess the midterm treatment outcomes of children with ICTEV who had been enrolled for treatment at Mulago National Referral Hospital in the period of 2006-2009.

**Methods:**

in November/December 2013, a cross-sectional study was conducted to assess the treatment outcomes of 68 feet of 45 children using the designed questionnaire and the PBS score; a pilot study of 10 neonates was performed prior to research. A good treatment outcome meant having a foot or feet that did not require any major or minor surgery.

**Results:**

forty-five (45) children with 68 ICTEV feet were evaluated; males 29 (64.4%) and 16 (35.6%) females with a mean age of 73.22 months (SD 11.364, range 48-96 months). Among the feet assessed, 46 (68%) had good to excellent outcomes while 22 (32%) had a relapse of moderate and severe deformity. Good functionality was seen in 61.8% out of which, 69% and 55.9% had no limitation in walking or running respectively.

**Conclusion:**

Ponseti treatment technique in children with ICTEV under the care of predominantly orthopaedic officers with some supervision from orthopaedic surgeons had fair to good midterm outcomes even in low resource settings like Uganda. Public health approach should be embraced in the management of clubfoot in Uganda by enhancing adequate comprehensive support supervision and establishment of reliable institutionalized systems for patient follow up which will lead to early detection and treatment of relapsed ICTEV cases or neglected clubfeet in the communities.

## Introduction

Worldwide 150,000-200,000 babies with idiopathic congenital talipes equino varus (ICTEV) are born each year and approximately 80% of them are born in the developing world with limited access to appropriate medical care. Untreated or incorrectly treated clubfoot soon becomes “neglected clubfoot” as the child grows older and learns to walk [1]. ICTEV is a common complex congenital anomaly in 0.39-7 children per 1000 births worldwide [2]. The affected children have abnormal foot anatomy and biomechanics with the affected feet being fixed in an extended, adducted position [3]. The burden of untreated or incorrectly treated ICTEV negatively impacts society and should be viewed as a public health issue so as to reduce its prevalence through early diagnosis and institution of appropriate treatment [4, 5]. The goal of treatment is to achieve early and full correction of all the four deformities of the foot, ensure that the patient has a functional, pain-free, plantigrade foot, with good mobility, without calluses, and does not need to wear modified shoes [6, 7]. The treatment modalities for ICTEV have evolved through a series of trials to include; conservative and surgical methods since the 18^th^ century.

Ponseti in the 1950´s developed a non-operative technique of management of ICTEV and made his publication in 1963, registering excellent outcomes in terms of function. The early treatment outcomes have kept improving since the time many surgeons began embracing the technique about 35 years later. Ponseti technique in the past 13 years has been embraced as the standard treatment for patients with ICTEV [8, 9] and it was only till May, 2005 that the Ministry of Health-Uganda (MOH) adapted it following the findings of Macharia J, which stated that, Ponseti technique of treatment could substantially reduce the disability burden caused by the ICTECV in Uganda [10]. They reported in their study that there were good clinical outcomes in 77.6% of the feet that were treated with Ponseti technique. This therefore yielded the conception of the Uganda Sustainable Clubfoot Care Project (USCCP) in March 2006 with a mandate of initiating a sustainable, universal, effective and safe treatment modality of ICTEV in Uganda based on the Ponseti method of treatment but later closed in 2010 with no sustainable follow up strategy for children with ICTEV [11].

The effectiveness of an orthopaedic officer delivered Ponseti care under the supervision of orthopaedic surgeons in Uganda was and is still unknown. We therefore wanted to assess this by looking at the midterm outcomes of treatment among the children with ICTEV and compare it with treatment outcomes of orthopaedic surgeons documented elsewhere. The likelihood of recurrence of the heel varus and forefoot adduction following the achievement of the deformity correction is of great concern. About half of these relapses can be attributed to neglected follow up and poor treatment compliance [12]. The implication of this undertaking was that, satisfactory outcomes would then support the Ministry of Health (MOH)-Uganda´s decision to embrace this mode of treatment which was based on the early treatment outcomes and if the outcomes were unsatisfactory, MOH would then consider a policy revision in this regard [11].

## Methods

A cross-sectional approach was used and evaluated the children at our Hospital´s club foot clinic located at the orthopaedic department of Mulago National Referral and Teaching Hospital, in the period of November and December 2013. We reviewed treatment records and extracted all the relevant data from the treatment register entered prior to the initiation treatment, such as: Pirani scores, number of tenotomies, casts needed to correct, availability of plaster of Paris (POP) duration of bracing, number of relapses, and treatment of relapses if it was documented as illustrated in the tables below.

The study population consisted of the following inclusion and exclusion criteria: 1) children that had been diagnosed with ICTEV; 2) enrolled for Ponseti treatment in the period of 2006-2009; 3) the child must have completed the four-year Ponseti treatment protocol that included bracing up to 4-years; 4) the child should have been formally discharged from the clinic following the assessment of the treating clinician that the deformity had achieved correction; 5) finally, the child would be in the follow up period of 4-8 years from the time he/she was enrolled into care; 6) children who did not meet the above inclusion criteria and had other forms of clubfoot such syndromic, positional deformities, and those who defaulted from the treatment protocol were excluded.

Selection of eligible patients was conducted using purposive sampling by use of the patient register having the names and contacts of patients who were treated in the clinic for ICTEV during the period of 2006 - 2009 and had completed the treatment protocol within the period of 2006 - 2009. Every caregiver of the patient was called upon to bring their child for clinical and radiological assessment till when the required sample size was obtained. For the caregivers or parents who were not in position to bring their children for the assessment, they were followed up by the principal investigator and the research Assistant in their respective communities. Using the Leslie Kish´s formulae for calculation of sample size for cross sectional studies,

$$N=\frac{Z_{\alpha }^{2}P(1-P)}{\delta^{2}}$$

Where N = sample size estimate of the number of feet of children treated for ICTEV by Ponseti method, P= Prevalence of good clinical outcome at mid-term evaluation. Using a prevalence of 95% in a study by Goksan (2002) [13] of treatment of club foot with the Ponseti method, N = (1.96)^2^x 0.95(1-0.95)/(0.05)^2^. N = 73 feet, as the sample size.

**Figure 1:**
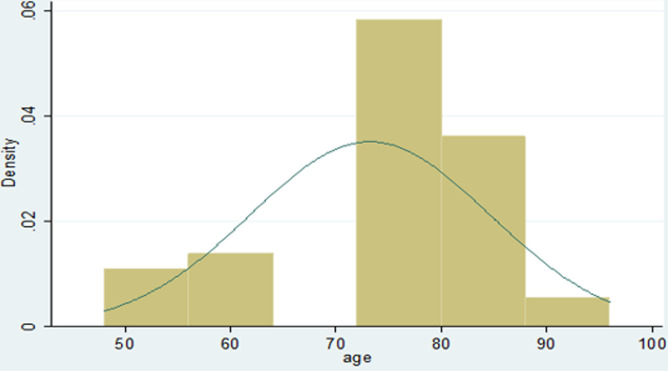
age distribution of patients with ICTEV assessed

**Figure 2:**
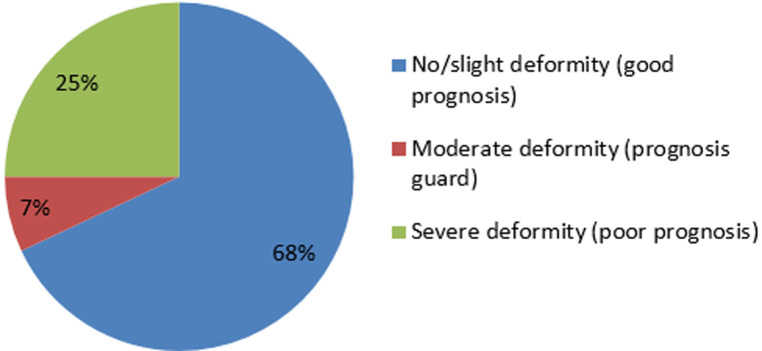
clinical outcomes of club feet treated by Ponseti technique

**Figure 3:**
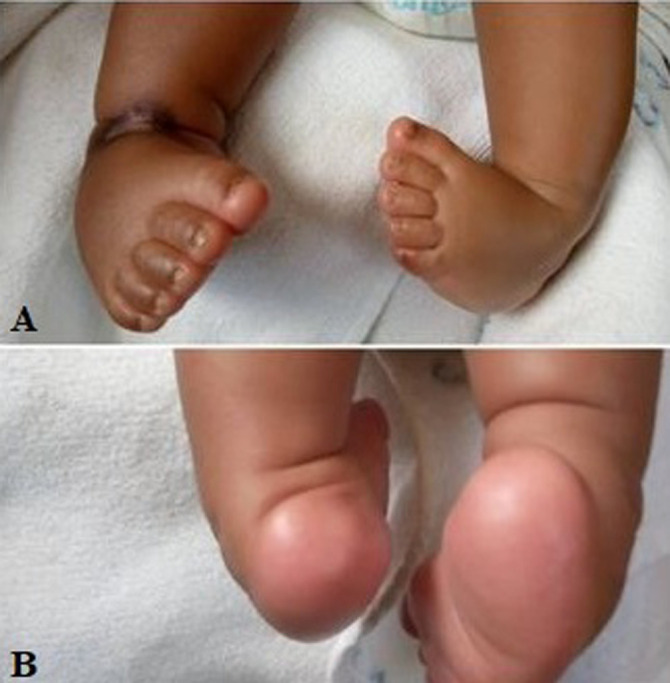
idiopathic congenital talipes equinovarus (ICTEV): A) anterior view; B) posterior view (bilateral)

**Figure 4:**
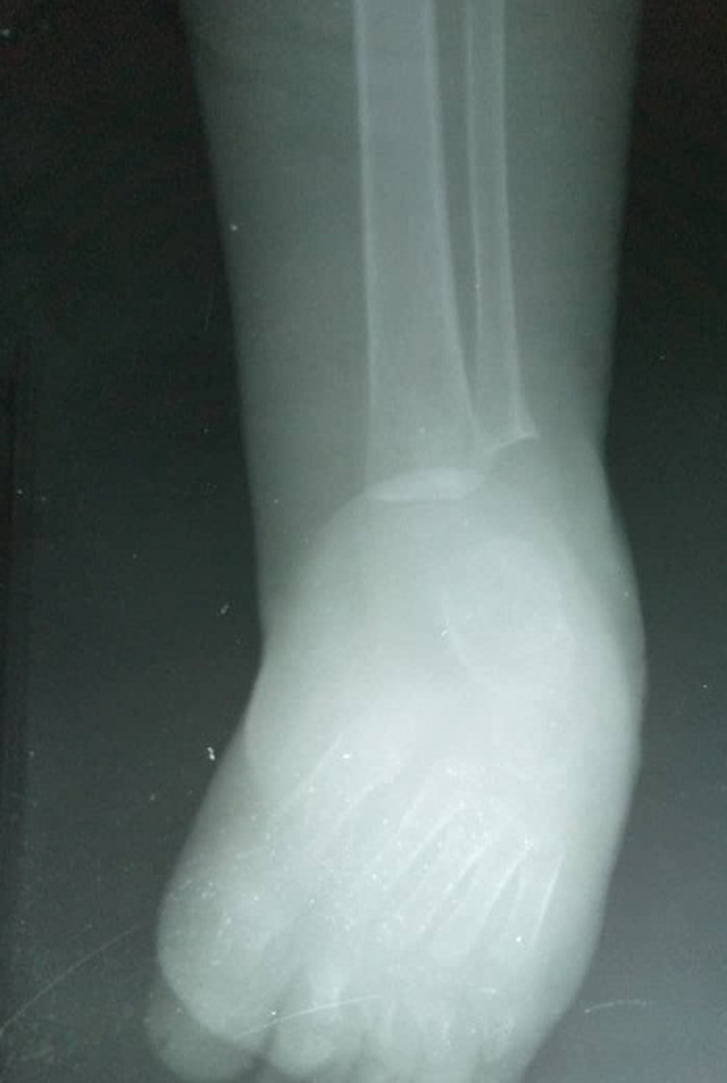
X-ray of RT distal part of the lower limb; note the lateral and medial malleolus of tibia and fibula respectively; the 3 cuneiforms, calcaneus, cuboid, navicular and talus

**Figure 5:**
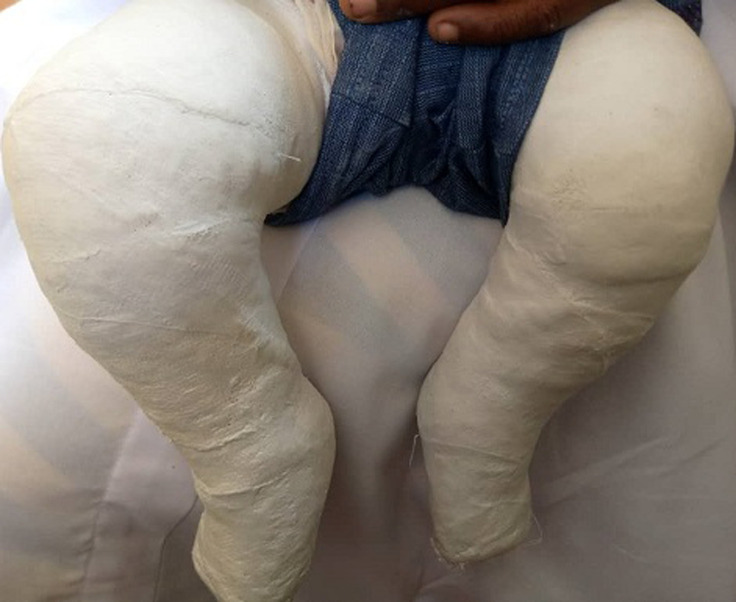
casting with plaster of Paris (POP)

However, we were only able to evaluate total of 68 feet, from 45 patients diagnosed with ICTEV in the period of 2006-2009, and in the follow up period of 4-8 years who had completed the treatment protocol. A pilot study of 10 neonates (n) averaging 10 months in age are included in photo-macrographs (Figure 1, Figure 2, Figure 3, Figure 4, Figure 5) attached below as an appendage.

### Measurement of the clinical outcome

The PBS (Shafique Pirani, Stephanie Boehm and Marc Sinclair, designed this clinical assessment tool of deformity in the Recurrent Clubfoot Following Ponseti Treatment) score was used to assess the clinical outcome of each affected foot. The tool had 7 signs that were assessed, hind foot varus, standing foot supination, dynamic foot supination, early heel rise, active dorsiflexion, passive dorsiflexion and passive subtalar abduction [14]. During the assessment of the hind foot varus, a patient in weight bearing position was examined from behind to assess heel alignment for varus/valgus and was scored as having varus or neutral/valgus deformity.

Passive dorsiflexion assessment was achieved by flexing the ipsilateral knee to 90° and putting the subtalar joint in a neutral position. The child´s foot was passively dorsiflexed above neutral and the outcome of that maneuver graded as normal, slight relapse, moderate relapse or severe relapse depending on the degree of dorsiflexion attained as >10°, 5°-10°, 0-5° and < 0° respectively. Subtalar abduction assessment was performed by flexing the ipsilateral knee to 90° and having the forefoot abducted with counter pressure on the head of the talus until resistance was felt. The angle of subtalar abduction was measured as that beyond neutral and was graded as > 10°, 5-10°, 0°, or less than 0°. Standing forefoot supination was assessed by having the child in a weight bearing standing position and examining the child´s forefoot from the front. Supination was either present or absent.

Dynamic forefoot supination was evaluated by observing the forefoot from the front as the child walked towards the examiner. In normal gait, all the metatarsal heads strike the ground at the same time. With dynamic forefoot supination, medial metatarsal head strikes the ground after lateral metatarsal heads. The assessment was whether the metatarsal heads struck the ground simultaneously or whether the medial metatarsal heads were delayed. This was scored as either present or absent. Lack of active dorsiflexion was assessed by flexing the child´s ipsilateral knee at 90°, with the subtalar joint in a neutral position. The child was then asked to actively (self) dorsiflex his/her foot maximally and the ability to do this above neutral was either graded as present or absent [15]. Early heel rise was assessed by observing the hind foot from behind as the child walked away from the examiner. In normal gait, heel strike was followed by a flat foot. Heel rise occurred only after the opposite foot had achieved heel strike. With early heel rise, the involved heel lifted off the ground before the opposite foot achieved heel strike. Ipsilateral heel rise was seen when the heel was rising before the contralateral heel achieved heel strike; it was either present or absent. Treatment outcomes were therefore categorized into: 1) the child having no or slight deformity, 2) moderate deformity and 3) severe deformity. Any child with no or slight deformity was considered as no relapse and those with moderate and severe deformity as a relapse case [15].

### Measurement of patient functionality

A disease specific instrument for idiopathic (DSI) clubfoot tool was used for assessment of parental satisfaction and functionality. Roye *et al*. demonstrated that the scale could be used as an overall measure of clubfoot treatment outcomes with two distinct subscales: function and satisfaction [16].

### Data management

Data was collected with the help of two research assistants who had undergone a three-day training to equip them with appropriate skills of collecting data using the questionnaire and PBS tool. Information on Initial Pirani score and feet with tenotomy were collected through interviews and from the patient cards. The collected data was then double entered to the Epi-info version 3.1. to ensure correct data correctly which then went through a second level of checks and editing before being exported to STATA version 12.0 for analysis.

### Data analysis

STATA 12.0 software was used for the analysis of the data exported from EPI-info database designed specifically for the study. An analysis design consistent with the EPI-info database was prepared so that the data is exported directly into the STATA database making it compatible and ready for data cleaning and analysis and descriptive analysis was done to generate frequencies and proportions.

### Ethical considerations

Ethical clearance for this study was sought from the Orthopaedic Department, Mulago Hospital, the School of Medicine, Ethical Review Committees, and the Uganda National Council of Science and Technology (#REC REF 2013-1380). Written informed consent was obtained from the patient´s parent or guardian following a clear explanation of the study and were enrolled with assurance of their comfort, privacy and confidentiality. To the benefit of the client, those found to have uncorrected deformities had to be enrolled for appropriate non-surgical or surgical intervention. Awareness creation on the need of follow up was done since there is likelihood of recurrence of the heel varus and forefoot adduction after achievement of correction. Half of these relapses could be attributed to neglected follow-up and poor treatment compliance [12].

## Results

Total of 45 patients (68 feet) were assessed with 23 patients having bilateral foot involvement. Their initial Pirani scores ranged from 3-6, a majority of whom 37 (54.4%) had an initial severity Pirani score of 6 at the time of enrollment, 82% (56 feet) needed tenotomy at the final stage of treatment and 16.2% (11 feet) of the feet needed additional surgery (TATT and PMR). The mean number of casts changed to achieve clinical correction was 5.7 (SD 3.236) as shown in Table 1.

**Table 1:** characteristics of the club feet assessed

Characteristics	Frequency (n=68 feet)	Percentage (%)
**Feet involvement**		
Unilateral	22	32.4
Bilateral	46	67.6
**Foot affected**		
Left	31	45.6
Right	37	54.4
**Initial Pirani score**		
3	2	2.9
4	11	16.2
5	18	26.5
6	37	54.4
**Feet with tenotomy done**		
Yes	56	82.4
No	12	17.6
**Feet with additional surgery done**		
Yes	11	16.2
No	57	83.8
**Mean number of tenotomies performed**	1.3	SD 3.236
**Mean number of P.O.P casts applied**	5.7	SD 0.681

### Socio-demographic characteristics of patients enrolled in the study

**Figure 6:**
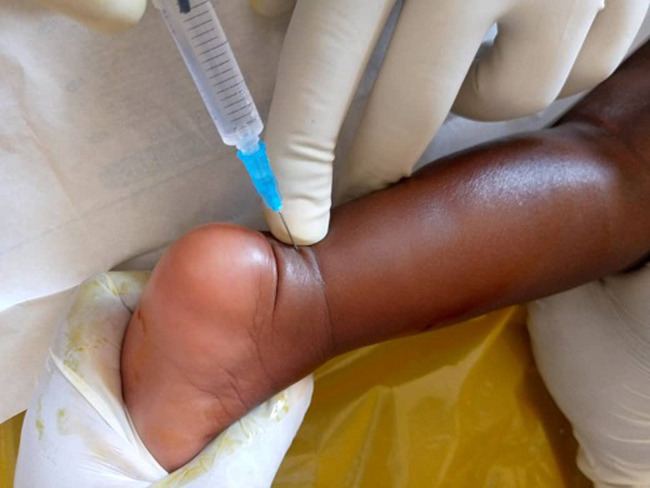
ameliorative injection procedure

A total of 45 patients with ICTEV were studied, 29 (64.4%) males and 16 (35.6%) females, with a male to female ratio of 1.8: 1 (Table 1). The mean age of the patients studied was 73.22 months (SD 11.364, range 48-96 months). 39 patients (86.7%) presented early for treatment while 6 (13.3%) presented late. Overall, there were more children who had been enrolled for treatment earl than those who enrolled late. The age distribution of participants involved in the study is shown in Figure 6. The minimum follow- up evaluation age was 4 years (48months) with a mean age of 6.1 years (73.2 months). The children´s ages ranged from 4-8 years as indicated in our patient age range.

### Clinical outcomes

**Figure 7:**
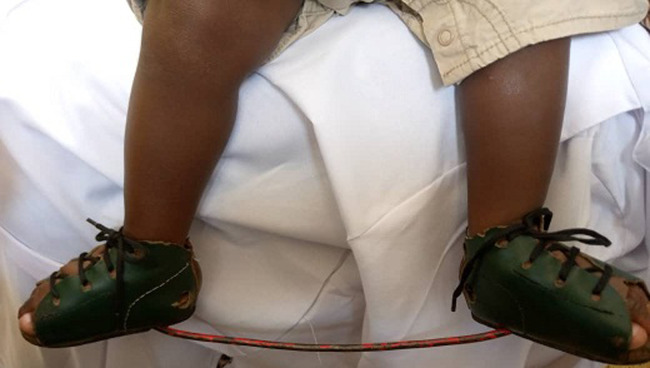
photo of (bilateral) steenbeek foot abduction braces (SFAB)

In the assessment of the 68 feet treated with the Ponseti method for ICTEV, 46 feet (68%) were found to have no deformity; good to excellent treatment outcomes and thus a good prognosis while 17 feet (25%) had severe deformity and 5 feet had moderate deformity as shown in Figure 7.

### Functional outcomes

The functional assessment of the feet of children treated for ICETV was done using the DSI tool developed by Roye *et al*. in 2001 [16]. Various parameters of function were evaluated to include aspects of gait, extent of limitation during limb use and presence of pain as shown in Table 2 below. Among the patients who were treated and evaluated in the midterm, 61.8% had never reported an episode of pain in affected foot. A good proportion of children, 69% and 55.9% reported to have no limitation in their ability to walk or run respectively. Parameters used for assessment of functionality of the treated feet are shown in Table 2.

**Table 2:** parameters used for assessment of functionality of the 45 children

Functional features assessed in children treated for ICTEV	Percentage (%)
**The child to ever complain of pain in his/her affected foot**	
No	61.8
Yes	38.2
**The child being limited in his or her ability to walk**	
Not at all limited	69.1
Somewhat limited	22.1
Moderately limited	8.8
Very limited	0
**The child being limited in his or her ability to run**	
Not at all limited	55.9
Somewhat limited	32.4
Moderately limited	8.8
Very limited	2.9
**The child to complain of pain during heavy exercise**	
Never	63.2
Sometimes	17.7
Usually	7.4
Always	11.7
**The child to complain of pain during moderate exercise**	
Never	77.9
Sometimes	11.8
Usually	4.4
Always	5.9

## Discussion

The main objective of the study was to determine the midterm clinical, functional and radiological outcomes of patient in Mulago hospital-Uganda treated for ICTEV using Ponseti technique. The majority of children with ICTEV attending club foot clinic at Mulago Hospital are managed non-operatively using the Ponseti technique. Ponseti technique has now been used widely for the past 7 decades since 1950s and has been proven to be very effective up to 98% [17]. The clinical outcome was evaluated using the PBS tool (Pirani *et al*.), developed for specifically assessing the ambulant group of children with ICTEV. The radiological outcomes were assessed by use of plain radiographs obtained from patients using the Beatson and Pearson method of radiological foot evaluation.

### Clinical outcomes

A total of 68 feet were assessed, out of which 46 feet (68%), had good clinical outcome with no or slight deformity while 22 feet (32%) had poor treatment outcome with moderate and severe deformity. The findings of our study (Figure 6 and Figure 7, Table 1 and Table 2) show a relatively low clinical outcome as compared to findings by other authors who have reported excellent treatment outcomes that have ranged from 82.2% to 98% [17-19]. However, a study conducted in Brazil middle income countries, reported a clinical outcome of 66.6% [6]. The results (Figure 6, Figure 7) in our study 68% are close to this because low income countries face similar problems. Poor treatment outcomes were recorded in 22 feet (32%) of the feet studied. Among these feet with relapse, 17 feet (25%) had severe deformity thus contributing to a high proportion of patients with poor prognosis and requiring further treatment.

Other studies (Table 1, Table 2) show a relapse rate of 10% [17] unrelated to age of presentation or severity of the deformity but was related to noncompliance to bracing and 6% rate residual deformity among patients with ICTEV treated by Ponseti Method [20]. However, the results of this study are close to those of the study in India of midterm evaluation of clubfoot treated with the Ponseti method that showed a relapse rate of 28.57% [19]. Uganda and India face almost similar factors.

Steinman *et al*. [21] compared the Ponseti and the French functional method of treatment of ICTEV finding initial correction rate at 94.4% for the Ponseti, and relapses up to 37% with the Ponseti method, similar to the findings in this study. A few studies have attempted to classify relapse among patients being treated by the Ponseti method but there is still no universal language of distribution of relapse pattern [20].

It´s very important to note that early identification of relapse and its early intervention would prevent necessity for major soft tissue surgery. A poor treatment outcome has huge financial implication on the health care system to both the patient and family. The treatment required to correct a relapsed clubfoot will require non-operative and operative modalities which is costly in terms of time, monetary values, physical, psychosocial stress/ strain to the patient and family. As a result, many patients end up with neglected deformity causing persistence of clubfoot and physical impairment in the affected child.

### Functional outcomes

In this study 61.8% of the patients reported no pain, an evidence of good functional outcomes associated with the Ponseti conservative method of treatment compared to the surgical soft tissue release. This was evident in a study conducted by Dobbs [22] where out of 73 clubfoot that underwent surgery 56% had moderate to severe pain due arthrosis. As much as the surgically treated foot would look cosmetically good following soft tissue releases, and osteotomies, its long-term functional outcomes are poor compared to those managed by Ponseti methods.

## Conclusion

Ponseti at midterm evaluation still has good treatment outcomes in Ugandan settings and implies that many children with ICTEV have been transformed to better life styles. There is however a need to undertake a monitoring and evaluation approach so as to intervene dropping rates of treatment outcomes by the treating clinicians and the Ministry of Health at large. This is critical because, the initial early treatment outcome was at 77.6% appealingly providing good hope for the future children with ICTEV deformity in Uganda. The observation in this study therefore calls for a critical system analysis in the path of clubfoot treatment health care delivery to the community in order to establish sustainable interventions so as to attain quality service delivery with excellent treatment outcomes in the country. The functional outcomes are equally good with the majority of patients reporting no pain, being able to wear shoes of their liking and having no limitation during walking or running. This is a sign of an improved life style and quality of life and correlates with good patient and caregiver satisfaction. This study therefore concludes that, the Ponseti method of treatment is still the most effective method of treatment with good to excellent treatment outcomes in the midterm. In our settings, however, more strategic planning is required to improve the outcomes. It is therefore necessary to identify and provide early intervention on the relapse cases. This can be achieved through comprehensive patient follow up plan in order to minimize cost of neglected clubfoot management and maximize patient´s clinical and functional outcomes. Recommendations: 1) Strategies to increase on community awareness and sensitization about clubfoot deformity and its complications should be taken up by the Ministry of Health Uganda and consider clubfoot in the context of public health. This would improve health care seeking practices leading to the reduction in the prevalence of neglected clubfeet in Ugandan communities. 2) The study recommends that there should be early identification of relapsed cases and early intervention by the treating physicians. 3) A study should be done to determine the factors that influence the treatment outcomes both in the midterm and long term such that factors that are associated with good outcomes are promoted in the community.

### What is known about this topic

- It was established, the Pirani, Boehm and Sinclair (PBS) tool in assessment and treatment of clubfoot had a combination of plantar angular dynamic foot supination, passive subtalar abduction and active dorsiflexion among other movements for congenital talipes management;
- Previously, there has been a total overestimation statistical subset of foot deformity in middle income countries [19], post-treatment relapse from several studies was pegged at 34.03%.

### What this study adds

- We can confirm a small fraction of children can still have foot structural complications after following known Ponseti method to completion; our study recommends cautious lengthening of the Achilles tendon for toddlers above 22 months, if percutaneous Achilles tenotomy fails;
- Procedure Achilles lengthening under general anaesthesia takes about 60mins to complete; ultimately allowing the child's foot to stretch and grow into the right position. Our documentation suggests acetaminophen for discomfort and a post-surgical cast from toe to thigh for 5 weeks until determination is made on veracity healed tendon;
- A more pragmatic and holistic data from our research demonstrates a possible relapse rate of 28.57% following applied modification of the Ponseti protocols.
